# An examination of the language construct in NIMH's research domain criteria: Time for reconceptualization!

**DOI:** 10.1002/ajmg.b.32438

**Published:** 2016-03-10

**Authors:** Brita Elvevåg, Alex S. Cohen, Maria K. Wolters, Heather C. Whalley, Viktoria‐Eleni Gountouna, Ksenia A. Kuznetsova, Andrew R. Watson, Kristin K. Nicodemus

**Affiliations:** ^1^ Department of Clinical MedicineUniversity of Tromsø−The Arctic University of NorwayTromsøNorway; ^2^ Norwegian Centre for eHealth ResearchUniversity Hospital of North NorwayTromsøNorway; ^3^ Department of PsychologyLouisiana State UniversityBaton RougeLouisiana; ^4^ School of InformaticsUniversity of EdinburghEdinburghUnited Kingdom; ^5^ Division of PsychiatryUniversity of EdinburghEdinburghUnited Kingdom; ^6^ Centre for Genomic and Experimental MedicineInstitute of Genetics and Molecular MedicineUniversity of EdinburghEdinburghUnited Kingdom

**Keywords:** RDoC, language, gene, speech, mental illness

## Abstract

The National Institute of Mental Health's Research Domain Criteria (RDoC) Initiative “calls for the development of new ways of classifying psychopathology based on dimensions of observable behavior.” As a result of this ambitious initiative, language has been identified as an independent construct in the RDoC matrix. In this article, we frame language within an evolutionary and neuropsychological context and discuss some of the limitations to the current measurements of language. Findings from genomics and the neuroimaging of performance during language tasks are discussed in relation to serious mental illness and within the context of caveats regarding measuring language. Indeed, the data collection and analysis methods employed to assay language have been both aided and constrained by the available technologies, methodologies, and conceptual definitions. Consequently, different fields of language research show inconsistent definitions of language that have become increasingly broad over time. Individually, they have also shown significant improvements in conceptual resolution, as well as in experimental and analytic techniques. More recently, language research has embraced collaborations across disciplines, notably neuroscience, cognitive science, and computational linguistics and has ultimately re‐defined classical ideas of language. As we move forward, the new models of language with their remarkably multifaceted constructs force a re‐examination of the NIMH RDoC conceptualization of language and thus the neuroscience and genetics underlying this concept. © 2016 The Authors. *American Journal of Medical Genetics Part B: Neuropsychiatric Genetics* Published by Wiley Periodicals, Inc.

## INTRODUCTION

The National Institute of Mental Health's Research Domain Criteria (henceforth RDoC) Initiative “calls for the development of new ways of classifying psychopathology based on dimensions of observable behavior” [Insel et al., 2010]. These dimensions are meant to reflect relatively specific domains of functioning that can be traced to a coherent mechanism across varying levels of human organization (e.g., genetic, molecular, physiological, behavioral). As a result of this ambitious initiative, “language” has been identified as an independent construct in the RDoC matrix, under the Domain Cognitive Systems. Although constantly evolving, as of late 2015, language is defined in the RDoC as “a system of shared symbolic representations of the world, the self and abstract concepts that supports thought and communication” [RDoC, 2015]. Language is measured across two broad “paradigms.” The first, language production, includes “naming” which involves verbal descriptions of visual depictions of events and states” and is often coupled with “linguistic corpus‐based analyses of language output”. The second, language perception, includes both “on‐line” measures, which are based on responses (e.g., listening, reading, eye movements) to verbal stimuli and “off‐line” measures which involve the ability to detect, distinguish, and answer questions about verbal features. Indeed, the focus on language is well‐deserved as language is a feature common to many different types of psychopathology, and can be affected in a myriad of ways. Examples include reduced speech production (e.g., alogia in schizophrenia or selective mutism in anxiety spectrum disorders), a restricted range of expression (e.g., flattened affect or psychomotor retardation), deficits in receptive language abilities (e.g., literal interpretation of proverbs in autistic spectrum disorder). Neuroimaging and genetic investigations of language in serious mental illnesses (SMIs) are starting to generate results that are tantalizing, although still in their infancy and require a standardized framework to be most effective. For these reasons, we contend that the advancement of a trans‐diagnostic framework for understanding language in SMI is a critical step toward understanding their pathophysiological mechanisms.

In reconceptualizing the RDoC framework, our first line of argument suggests that a substantially more comprehensive and multidisciplinary view of language is needed if an RDoC‐like construct is to be maximally useful in translational research of clinical disorders that affect cortical functions including language. Such a re‐definition necessarily must be drawn from the linguistic, speech, cognitive, and affective sciences, spanning basic articulatory processes to those involved in complicated social interactions, and thus will open more profitable avenues of research in genomics and neuroscience. Our second line of argument is that only with a reconceptualization of this language construct can we successfully leverage emerging technologies and methodologies, such as those involving automated computational linguistics or “big data,” large‐scale cohort studies (e.g., linguistic “biobanks”) where the benefit of these assays can be capitalized on by clinical studies and trials. A core premise of this article is that emerging technologies can be applied to efficiently, objectively, and unobtrusively obtain and evaluate language in both clinical and natural settings, and that the application of these technologies can be a boon for RDoC applications and, eventually, clinical assessment and treatment.

In this article, we ground language within an evolutionary and neurobiological perspective, and then review potential limitations to current measurements of language in studies of SMI. Next, we discuss recent genetic and neuroimaging investigations of language within the context of psychiatric disorders. In the final part of the article, we outline ways to reconceptualize language within the RDoC framework using state‐of‐the‐art approaches including: (i) novel modalities (e.g., acoustics, ultrasound), (ii) cutting‐edge computational approaches (e.g., machine learning, continuous language representations such as latent semantic analysis), and (iii) taking these approaches into large biobanking efforts.

## LANGUAGE FROM AN EVOLUTIONARY AND NEUROBIOLOGICAL PERSPECTIVE

The RDoC notion of language has its origin in a reductionist framework, and this begs the question as to the purpose that speech and language evolved to fulfil. Assuming their primary purpose is to communicate meaning, a framework emerges that includes a broad range of functions, such as fine‐tuned motoric gestures, episodic recollection, generativity, and lexical creativity. Furthermore, comparative neurology promotes a case for humans' incremental prefrontal expansion during evolution as reflecting a prolonged selection favoring an alternative to the more basic and highly effective correlative learning strategies, namely combinatorial and hierarchical learning [Deacon, 1988, 1992a], and it is assumed that symbolic acquisition was favored. Such a co‐evolutionary process of neurological adaptation and changes in language use [Deacon, 1992b] is compelling and, regardless of what any changes in language use may have been, the framework nonetheless advocates that symbolic communication be included in a definition of language. Clearly, this necessarily widens any definition of language to include specific forms of thinking and learning.

At the core of this argument are two issues. First any genetic changes in the evolution of the human brain that made it capable of language seems small (i.e., any difference between humans and non‐human primates in this matter seem relatively modest). Second is the question of whether these evolutionary changes resulted in any specific localization for such so‐called language functions. Traditionally, language has been associated with specific regions of the brain, notably Broca and Wernicke areas around the planum temporale in the left cortical hemisphere, yet lesion studies seem to suggest that the localization of language is somewhat plastic and indeed the data on localization remains quite controversial. When people recover language after a stroke other areas of the brain reorganize as function returns. Although the changes are focused in the homologous area of the other hemisphere and areas close to the damaged area [Cramer, 2008], this pattern reflects only the average changes and there is marked inter‐individual variation in brain reorganization after an insult that can include areas anatomically far removed contributing to recovering function. There are of course multiple levels to language and its evolution. Protolinguistic processes have been located in the subcortical and paleocortical brain and in the posterior neocortex (i.e., outside so‐called classic anterior parts such as Broca's) [Van Lancker, 1987]. Furthermore, a compelling case has been made for emotional vocalization within the limbic paleocortex being a legacy of the phylogenetic transition from reptiles to mammals [MacLean, 1988, 1990]. Moreover, it is widely assumed that the enlarged perisylvian region of the left hemisphere is central to language development and function [Geschwind and Galaburda, 1987], yet this asymmetry was also present in our ancestors *Homo erectus*. More problematic are findings that question whether the perisylvian association cortices, in toto, even have consistent asymmetry at all since whereas the planum temporale seems larger on the left, the planum parietale (its counterpart in the ascending ramus of the Sylvian fissure) is larger on the right ([Jancke et al., 1994]; for commentary see Elvevåg and Weinberger, 1997]). Neuroanatomical changes that were distinctly more unique to humans may have in fact been those within the prefrontal brain which opens the possibility for both an upstream and downstream evolution and ontogenesis of language and metacognition.

Following on the line of argument above, the centrality of symbols in language begs the question of where they might be “grounded.” Multiple possibilities emerge: first, these symbols (e.g., semantics and syntax) may emerge from visual and intermodal perceptual experience (i.e., from below). Second, it may be that semantics emerges from thinking (i.e., from above). Third, it is possible that social interaction is the medium for the emergence of semantic reference and syntax. Put differently, the cognitive system exists in a linguistically structured environment and has to conform to the environmental demands by learning to use the symbols and putting them together in suitable ways. Undoubtedly, this requires recognizing and using patterns. At a general level such a position is consistent with pluripotency of cortical computations, in this case “that the specificity of functions depends on the embedding of an area in the brain's connectome rather than on its intrinsic organization” [p. 329; Friederici and Singer, 2015]. Thus the computational principles employed in language are likely based on those that are also evident in other cognitive processes.

## CONCERNS WITH MEASURING LANGUAGE

Evolutionary and cognitive science perspectives notwithstanding, language is affected in a broad range of brain disorders, necessitating the development of tests of language that can be readily applied to heterogeneous populations in clinical settings. To date, there exists a large number of psychometrically “supported” measures of language; measures which vary tremendously in scope as well as whether they are based on self‐report [e.g., Raine, 1991], clinician impression [e.g., Andreasen, 1981], or objective performance [e.g., Gershon et al., 2013]; though the latter predominates clinical practice. Collectively, these measures have been instrumental for assessment and rehabilitation of clinical dysfunction in disorders stemming from neurodevelopmental or neurodegenerative conditions.

Not surprisingly, a large scientific literature exists supporting the use of these clinical tests, which assess abilities such as verbal fluency; spelling, reading, and writing skills; vocabulary; and receptive listening skills. While few of these tests were developed to understand SMIs per se, they have become the dominant method for understanding language in them. Verbal fluency, for example, which requires the generation of words given a set of specific parameters, has been the focus of several meta‐analyses in schizophrenia [e.g., Bokat and Goldberg, 2003; Henry and Crawford, 2005a] and in mood disorders [e.g., Henry and Crawford, 2005b; Bora et al., 2009].

From a psycholinguistic perspective, however, many components of language are not well represented in current clinical tests, and more importantly, from the extant literature. Indeed formal tests of language phonetics, prosodics, syntax, and semantics (beyond single word generation) are few and far between, except for the specific assessment of speech and language disorders. Consider further that major recent neuropsychological batteries either specifically developed for, or commonly used in SMI populations, exclusively measure language in terms of verbal fluency [Randolph et al., 1998; Keefe et al., 2004; Nuechterlein and Green, 2006]. While measures of other language abilities exist, for example, evaluating semantic aspects of language using the Thematic Apperception Test or the Rorschach, their use is controversial and normative data for this use has limited validity [e.g., Wood et al., 2003]. Thus, relying on existing tests provides a grossly inadequate view of language abilities or how we might measure them.

Moreover, many existing language tests have been designed to yield reliable measures for an epoch where results were based on the clinician's perception, and documented using available technology, namely pen and paper. Therefore, many of the tests and metrics that are employed today focus mostly on easily counted surface phenomena. Modern technologies allow for much more sophisticated measures of language. For example, when assessed by ear, intonation can only be described subjectively, whereas objective criteria, such as accuracy or reaction time, are overly simplistic. The alternative assessment through computerized acoustic analysis, as discussed below, allows acoustic features such as average pitch and pitch range to be automatically and objectively extracted, and described precisely [e.g., Jurafsky and Martin, 2008].

Beyond these concerns, it is also critical to consider that existing measures fail to account for contextual factors. The notion that language is a dynamic function that is dependent on a range of contextual factors has been foundational for modern linguistics and semiotics. Many situation‐specific factors affect how people use language to communicate, such as physical characteristics of the communication environment (e.g., background noise), the language used by the interlocutor [Pickering and Garrod, 2004], social factors relevant to the communication [Trudgill, 2000], and other idiographic factors. In addition, it is important to consider cognitive factors, such as attentional resources for producing speech while performing other functions (e.g., driving, walking) in order to better represent a more ecologically‐valid measure of language. Speech also differs as a function of automatic speech reception/production (e.g., counting from 1 to 10) versus more resource‐demanding speech (e.g., recounting specific childhood autobiographical memories). Context of use intersects with all the components of language described above. For example, irony detection *vis à vis* prosodic cues may fail due to problems on the part of the listener with social cognition or cultural unfamiliarity. A speaker with depression may fail to make the phonetic and prosodic adjustments that are required to be understood in a noisy environment [Lombard speech, c.f. Garnier and Henrich, 2014]. Importantly, existing clinical measures control for the potential impact of contextual variables by standardizing administration, and hence, miss the very opportunity to evaluate how language varies as a function of context. Moreover, the majority of extant language research in SMI has been reliant on these standardized measures, and thus, has systematically neglected the role of context in language. With these deficits in the state of current language measurement in mind, we turn our attention to current genomic, and then neuroimaging, investigations of language in SMI.

## THE GENOMICS OF LANGUAGE

Despite impressive advances in genetics technology, the measurable genetic effects on various cognitive constructs, including language, have thus far been modest. One limiting factor is the measurement of the cognitive constructs themselves. Thus, there is an urgent need for intermediate phenotypes that relate more directly to how genes affect neural systems and behavior [Green et al., 2008]. The approach of focusing on cognition as an intermediate phenotype in neuropsychiatric research is quite compelling [Goldberg and Weinberger, 2004; Meyer‐Lindenberg and Weinberger, 2006; Tan et al., 2008; but see Flint and Munafo, 2007]. The intermediate phenotype can be expressed in unaffected close (generally first‐degree) relatives of SMI probands on cognitive tasks. For many cognitive phenotypes the performance of close relatives is “intermediate” between probands and healthy controls, indicating the phenotype of interest is at least partially genetically influenced.

Importantly, such a research strategy is also advocated in the case of neurodevelopmental language disorders [Bishop, 2009]. In the quest to relate phenotype to genotype, a strong case has been made to move away from categorizing the disorder based upon clinical diagnosis to refining the cognitive phenotype [Newbury et al., 2005], and this is a hallmark feature of the RDoC framework. To illustrate, progress in research on neurodevelopmental language disorders has been reported by focusing on phonological short‐term memory, measured for example by nonword repetition. Such an approach to the phenotype does not assume that discovery of genetic relationships at this level (e.g., phonological short‐term memory) will indicate a gene for language, rather it reflects the realization that language and communication are composed of many building blocks which contribute in a quantitative fashion, which is naturally moderated by multiple alleles and environmental factors. In the case of phonological short‐term memory, it is arguably crucial in the acquisition of language [Gathercole and Baddeley, 1990]. Although research explicating the genomics of language in SMI is relatively modest, there exists evidence of genetic constraints that govern global language abilities, notably language acquisition and processing. Thus, any possible candidate “language gene” (e.g., FOXP2 [Lai et al., 2001]) most probably influences factors such as domain‐general procedural systems and genes that are downstream (e.g., CNTNAP2), the latter which modulates phonological short‐term memory [Fisher, 2006; Vernes et al., 2008; Whitehouse et al., 2011].

Following on from this reasoning, a compelling case has been made for rule‐ and memory‐based processes underlying language, such that the processing of rule‐governed knowledge (including syntax) is assumed to recruit the procedural memory system rooted in frontal/basal‐ganglia circuits, whereas the processing of memorized idiosyncratic knowledge (which includes “semantics”) depends on temporal lobe regions involved in declarative memory [Ullman, 2001, 2004]. This argument illustrates the complex and intertwined nature of episodic memory and the semantic and syntactic aspects of language. In sum, language abilities likely take contribution from a range of genetic mechanisms, as is typical of any complex trait. To explore this further, we consider language production and perception separately in the following sections.

### Genomics and Heritability of Language Production

As mentioned previously, much of the existing research investigating the genetics of language has been dependent on relatively circumscribed tasks that tap only basic processes (e.g., “naming”), and thus are not likely to capture all or even most of the relevant information that can be derived using more modern approaches. Another limitation in these studies derives from the modest sample sizes often employed by genetic investigations of language. However, with the advent of multiple very large‐scale biobanking efforts worldwide (e.g., UK Biobank [Collins, 2011], Generation Scotland [Smith et al., 2006]), we argue that state‐of‐the‐art approaches now have an unprecedented opportunity to be leveraged with adequate sample sizes to investigate the genetic architecture of language in individuals with and without SMI.

Verbal fluency tasks have been the mainstay of language production tasks in studying the genomics of SMIs. The reason for the focus on these tasks is that they are simple to administer and so have been widely used. Subsequently, verbal fluency performance has been shown to be heritable [e.g., Vandenberg, 1962; Bratko, 1996; Aukes et al., 2008]. In bipolar disorder, individuals currently in manic or mixed episodes who carried the catechol‐O‐methyltransferase (COMT) Val158Met G (Met) allele showed improved verbal fluency performance [Soeiro‐de‐Souza et al., 2012]. The COMT gene has been associated with numerous cognitive processes, including intelligence, executive functioning, working memory, and attention [for a review, see Dickinson and Elvevåg, 2009]. In patients with schizophrenia and controls, single nucleotide polymorphisms (SNPs) in the genome‐wide significant [Schizophrenia Working Group of the Psychiatric Genomics Consortium, 2014] gene TCF4 were significantly associated with verbal fluency (again, the number of words produced; individual group comparisons) [Wirgenes et al., 2012].

### Genomics and Heritability of Language Perception

Perception of language in SMI comprises a broad range of abilities, including judgements about semantic, syntactic, prosodic, and lexical factors of speech as well as social cognition. Deficits in many of these abilities have been well documented in a range of SMIs [e.g., Lavoie et al., 2013], and the few studies that exist often suffer from small sample sizes. In this section, we have expanded the discussion to also include a review of rarer disorders with highly penetrant mutations that effect language perception and comprehension.

Thus far, very few studies have been performed to examine the heritability or genetics of language perception, and those that do focus on relatively constrained facets of language, often exclusively on non‐verbal emotion or affect recognition. In general, existing studies support the notion that social perception and emotion recognition are poorer in biological first‐degree relatives of patients with SMIs. A recent meta‐analysis of 29 studies found moderate effect sizes in healthy first‐degree family members of patients with schizophrenia across a range of social cognition tasks, notably tapping mentalizing, emotional processing, and social perception abilities [Lavoie et al., 2013]. Allott et al. [2015] reported deficits in recognition of anger and surprise in first‐episode schizophrenia patients and a similar deficit in healthy first degree relatives of patients versus controls. However, as in many studies employing novel language‐related phenotypes, the sample sizes were relatively small (N patients = 30, N first‐degree relatives = 27, N controls = 30). A similar study design comparing autism spectrum disorder probands (N = 90), their unaffected siblings (N = 79), and healthy controls (N = 139) reported that autism spectrum disorder probands showed significant deficits in recognition of affective prosody versus healthy controls [Oerlemans et al., 2014]. Similar to results of the study of schizophrenia probands, siblings of autism spectrum disorder probands performed less well in the detection of emotional prosody versus controls, supporting the notion that nonverbal language deficits may be an intermediate phenotype for these SMIs. Ronald et al. [2005] reported high heritability in individuals with autism spectrum disorders in social cognition, with h^2^ estimated at >0.6. Irony perception and comprehension, a component of social cognition and of meta‐cognition more generally, is an important social skill that aids understanding between people which is often less fine‐tuned in patients with SMIs and autism‐spectrum disorders Emerging evidence is suggesting that this ability is modestly heritable [h^2^ = 0.27; McGrath et al., 2009].

Outside of SMIs, other, generally rare, disorders with highly penetrant mutations show deficits in speech and language. Williams syndrome is characterized by heart disease, failure to thrive, speech and language delay, and other cognitive deficiencies, but these individuals show exaggerated social behavior [Mervis and Shelley, 2011]. Williams syndrome is caused by a 7q11.23 deletion. Individuals with Williams syndrome show relatively preserved speech and facial recognition versus individuals with SMIs such as autism [Bellugi et al., 1999], making this syndrome of potential importance in disentangling the genetics of language delay versus understanding of social language processes.

Worthey et al. [2013] studied childhood apraxia of speech using an auditory perception task of verbal and nonverbal aspects of linguistic stimuli and found that poorer performance was observed in those carrying a potentially deleterious variant in one or two of the following genes: *FOXP1*, *CNTNAP2*, *ATP13A4*, *CNTNAP1*, *KIAA0319*, and *SETX*. Fronto‐temporal lobar degeneration and progressive non‐fluent aphasia are commonly associated with mutations in C9orf72. Rohrer [2010] reported that patients with non‐fluent aphasia performed significantly poorer than healthy controls on tests of acoustic (pitch, intensity, and duration of sound), linguistic (stress, e.g., “black and blue;” and intonation, e.g., “apple” versus “apple?”), and emotional prosody (recordings of neutral phrases like “one hundred and thirty‐seven” read out with different intonations to convey six basic emotions).

The brain degeneration condition in Huntington's disease is caused by mutations in the *Huntingtin* gene. Patients with this gene who have not developed signs of overt neurodegeneration show decreased performance on prosody comprehension tasks with performance comparable to that of stroke patients [Speedie et al., 1990]. Similarly, Vogel et al. [2012] collected speech samples from 30 *Huntingtin* gene mutation carriers and 15 unrelated healthy controls. Analyses of the acoustic properties showed that carriers spoke significantly more slowly than controls, with longer pauses between and within phrases and took longer to pronounce words.

### Ways Forward: Genomics of Naturalistic Language and Computational Approaches

Technological and methodological advances in objective analysis of language afford more sophisticated and ecologically valid tools for understanding the genetics of language. These advances will be discussed later in this article, but it is worth noting they are beginning to be applied to genomics research. Of note, using novel speech intermediate phenotypes derived from Latent Semantic Analysis (LSA) of performance on a category fluency task, Nicodemus et al. [2014a] showed that a measure of the unusualness of speech (the average vector length) produced in a one‐minute response to the cue “animal,” was significantly associated with a functional SNP in the gene Disrupted in Schizophrenia 1 (*DISC1*) in both male probands with schizophrenia and in male controls. Individuals who were minor allele carriers at rs121133766 produced significantly less complex terms in response to the cue “animal” than homozygous major allele genotype carriers. Although this is an intriguing initial investigation into the use of computational language phenotypes, these results are preliminary, and these computational approaches have not yet been shown to be heritable nor have they been studied at the level of GWAS or exome/whole‐genome sequencing, although we argue this is the logical next step in the study of the genomics of language.

## NEUROIMAGING OF LANGUAGE

Neuroimaging is another methodology that provides insights into the neurobiological mechanisms supporting language dysfunctions in SMI, and has a large literature involving language tasks. Investigations involving a variety of paradigms have been conducted using a broad range of tasks, including traditional verbal fluency (either overt or covert) tasks, lexical decision, semantic processing, speech comprehension and perception, prosody, paradigms exploring the understanding irony and metaphors, and more recently naturalistic language processes. In this section, we focus on functional magnetic resonance imaging (fMRI) as the dominant modality in the study of language in SMIs, though of course other neuroimaging techniques are important. The literature using fMRI alone is voluminous and hence cannot exhaustively be reviewed here. Rather, our goal is to provide a flavor of the types of tasks and methods that have been used, with a focus on their limitations when paired with neuroimaging as well as the evolution of more naturalistic and ecologically valid paradigms and analyses employing computational linguistic analysis approaches.

### Neuroimaging of Language Production

Due in large part to restrictions on physical movement (and speaking) within the neuroimaging scanner, research involving language production (see below for elaboration), particularly involving extended or “natural” speech has been limited. Verbal fluency is the most commonly used language production task in psychiatric neuroimaging studies, and is consistently associated with activation in the left middle and inferior frontal gyri, the cingulate gyrus, as well as the right cerebellum and the temperoparietal cortex [Stuss et al., 1998; Fu et al., 2002]. A recent meta‐analysis indicated both phonemic and semantic verbal fluency tasks activated these regions, but there was potential spatial separation in frontal sub‐regions (left inferior frontal gyrus) depending on which type of verbal fluency task was performed [Wagner et al., 2014]. Although there is a degree of inconsistency, in patient populations, particularly schizophrenia, there is generally reported to be additional recruitment of brain regions in order to establish performance at a similar level to healthy controls, usually involving right‐sided homologs of what are considered left lateralized language regions [Costafreda et al., 2011]. Typically, similar findings are also reported in individuals at high familial risk [Li et al., 2007a] as well as in bipolar disorder, although to a lesser degree than in schizophrenia [Costafreda et al., 2011].

### Neuroimaging of Language Perception

A broad range of language perception and comprehension abilities in SMI have been examined. For example, a relatively large literature focusing on language perception of semantic and lexical features at the “word” level has emerged. Of note, studies involving discrimination between word and non‐word stimuli [i.e., lexical decision tasks; e.g., Li et al., 2007b; Natsubori et al., 2014; Sass et al., 2014a,b], are common. In healthy participants, such tasks have consistently shown stronger word than non‐word activity in widespread left lateralized regions, and greater non‐word activity associated with inferior frontal regions [Natsubori et al., 2014]. Although no unequivocal consensus of findings exists, typically this leftward lateralization of brain activity related to lexical decision and speech processing has been reported to be significantly reduced in patients with schizophrenia compared to controls [Sommer et al., 2001, 2003; Ngan et al., 2003].

Affective perception involving language have also been examined [Mitchell et al., 2004; Eigsti et al., 2012]. As with many emotion paradigms, the processing of the emotional content is indirect, where participants are asked to make some judgement about the content of the speech and not explicitly requested to attend to the affective component. These types of task, like other language‐based paradigms, have not generated a coherent pattern of deficits in SMI populations. However, these studies have indicated an increased recruitment of higher‐order cognitive regions in the processing of emotional prosody in autism [Eigsti et al., 2012], as well as decreased fronto‐temporal connectivity, and increased right lateralization in schizophrenia [Mitchell et al., 2004; Leitman et al., 2011].

The neural bases of higher order language perception abilities have also been examined. For example, linguistic irony, which is a metacognitive and social cognitive function, is reported to be disrupted in many psychiatric conditions, including schizophrenia and autism [Rapp et al., 2010; Rapp et al., 2013; Varga et al., 2013]. These studies have indicated involvement of posterior medial prefrontal and right temporal regions in the defective irony comprehension in schizophrenia, together with an association between activation in these regions and schizotypal personality traits [Rapp et al., 2013].

### Limitations of Neuroimaging Methodologies in the Study of Language

A discussion of the use of neuroimaging, particularly functional magnetic resonance imaging (fMRI) in the study of linguistic processing in SMI, would not be complete without some mention of the difficulties and limitations of such paradigms within the scanner environment. As mentioned above, one of the main problems with neuroimaging is the need to minimize movement during the experiment. The generation of speech inside the scanner creates motion artifacts and susceptibility changes in and around the vocal cavity which can fundamentally confound analysis. Several methods have been employed to overcome these issues including the internal generation/articulation of responses [Curtis et al., 1998; Curtis et al., 2001; Boksman et al., 2005; Takami et al., 2007], or the use of continuous paradigms where individuals have a set time period to generate as many words as possible [Weiss et al., 2004; Backes et al., 2014]. The inherent problem with both paced and continuous paradigms is the inability to monitor the behavioral responses of the participant within the scanner. A number of functional neuroimaging paradigms have more recently utilized techniques such as sparse temporal sampling or clustered volume acquisition, that allow for overt response generation between periods of image acquisition hence enabling ongoing monitoring of behavioral responses [Fu et al., 2005; Curtis et al., 2007; John et al., 2011; Allen et al., 2012; Backes et al., 2014]. Other methodological techniques have been employed to overcome these difficulties at both the data collection and analyses levels [Crosson et al., 2007].

Another important caveat to functional neuroimaging is that the results are based on the premise of cognitive subtraction. The measured activation is a representation of the relative differences in brain activity between two or more brain states elicited by the task and therefore dependent on the intricacies of not only the task but also “baseline” conditions. Individual differences in default network activation, and their relatively importance to language functions, have yet to be well understood. Hence, language paradigms, falling into discrete categories as defined by psychological constructs, should be interpreted cautiously as to whether the subtracted component truly isolates the discrete function of interest.

In addition, performance matching of patient populations to comparison groups is another consideration for the neuroimaging literature, conducted in order to interpret findings as relating to disease‐specific differences in neurobiology rather than simple behavioral disengagement from the task. Fedorenko et al. [2012] have further argued that the lack of consistency between language studies may also be due to differences in anatomical and functional specialization among individuals and that conventional analysis methods may actually hinder knowledge about the functional architecture of the language system. Furthermore, in patient populations, the fundamentals of functional specialization frequently observed in healthy individuals may be less applicable.

### Ways Forward: Neuroimaging of Naturalistic Language and Computational Approaches

As with genomics, technological and methodological advances in objective analysis of language have afforded unique opportunities for neuroimaging of language (discussed later). These advances are beginning to be seen in some recent studies. For example, a recent study examined the relationship of computationally derived semantic coherence scores from free discourse generated outside of the scanner to activation during a word monitoring task performed inside the scanner [Tagamets et al., 2014]. While coherence scores from free discourse in healthy individuals were related to executive function regions, the coherence scores in patients with schizophrenia were related to auditory and visual regions, particularly superior/middle temporal cortex. Of course, the limitation of this study is that these two correlated measures were acquired at two different time points and using two different tasks (free speech versus task performance on a simple cognitive task). Beyond the use of computational methods to understand language, this study is important in that it focused on language discourse, which is an inherently more ecologically valid method of examining language production compared to verbal fluency tasks.

Examples of using ecologically valid methods for understanding language perception are many. Of note, there are an increasing number of studies exploring the brain's typical functional responses to continuous, naturalistic, and dynamic natural stimuli, including speech [e.g., Silbert et al., 2014; Fang et al., 2015]. Although considered more ecologically valid in comparison to traditional focused task based methods, this type of approach has created methodological challenges including the modelling of the stream of external stimuli and the corresponding brain responses. One such study used narrative shifts in story listening as the cue to examine the corresponding neural responses and reported the involvement of the precuneus and posterior cingulate in updating mental story representation [Whitney et al., 2009]. Silbert et al. [2014] measured activation during the telling of a rehearsed real‐world narrative within the scanner, and the audio playback of the original story to the listener, thus capturing both production and comprehension components of speech. The data indicated that speech production recruits an extensive bilateral network of linguistic and extralinguistic brain areas, in line with current conceptualizations of the involvement of widespread networks in these processes. In another example, Sabb et al. [2010] attempted to predict symptomatic and functional outcome in adolescents at high risk for psychosis using a naturalistic task to assess the ability to comprehend discourse. The task here involved evaluating question and answer pairs based on either the topic and or the semantic logic of the sentence [Sabb et al., 2010]. The at‐risk participants demonstrated increased neural activity in language‐associated brain regions, proposing finding interpreted as indicative of neural inefficiency in those at greatest risk for psychosis.

At present, there is a very limited neurobiological understanding of how various aspects of language are integrated—for example, in how words are combined and meaning is created, although recent research on the spatiotemporal dynamics of meaning construction is extremely promising [Bemis and Pylkkänen, 2012; Pylkkänen et al., 2014]. Studying the dynamics of creating coherence is a valuable research framework that enables detailed examination of where problems in generating meanings may arise in patients with schizophrenia [Ditman and Kuperberg, 2010], and may benefit from the use of MEG, event‐related potentials (ERP), and other technologies with high temporal resolution. Arguably paradigms with such superior temporal resolution hold the clues for why, for example, those with a genetic risk for schizophrenia (by virtue of having a higher familial risk for the disorder than the general population) have been shown to display a pattern of hemodynamic modulation in inferior frontal/temporal network as a response to a simple word task that seems very different to the activation spread from a control group [Thermenos et al., 2013].

## RECONCEPTUALIZING LANGUAGE FROM AN RDoC PERSPECTIVE

It should be clear to the reader that language is a complicated and multifaceted construct. The notion that language is a unitary construct, as proposed by RDoC, is overly simplistic and the classic ideas of language functions being relegated solely to specific regions of the left temporal lobe is simply outdated in the modern neuroscience era. Indeed, “the era of the classical model is over” (p. 14125; Poeppel et al., 2012]. Associated with this neuroscience‐based re‐conceptualization of language are radical improvements in resolution, both in terms of experimental techniques that afford superior spatial and temporal resolution but also in terms of methodological sophistication because of the increasing dialog between the different levels of research. Within language research a paradigm shift is occurring courtesy of the explosion of cross‐disciplinary research and significant improvements in technological and conceptual resolution. This article is in keeping with this philosophy, namely that the RDoC construct of language is in need of a major reconceptualization. Moreover, despite ambiguity regarding its roots, language is clearly intertwined with a host of other cognitive and socioemotional abilities and functions. Indeed, the RDoC authors acknowledge this, as documented by the Cognition Workshop Proceedings [RDoC, 2015]: “Language involves a mapping between thought (production) and sensory representations (comprehension) via a symbolic system of multiple representations (which include prosody, phonology, syntax, orthography, and lexical‐semantics).” Interestingly, many language‐related constructs are included within the RDoC matrix as distinct constructs. For example, “Social Communication,” “Production of Non‐Facial Communication,” “Reception of Non‐Facial Communication,” “Self‐Knowledge,” “Perception and Understanding of Others,” and “Understanding Mental States.” Whether this taxonomy of constructs is optimal or useful in future research is unclear.

That being said, a core question at hand concerns whether the language construct, as currently conceptualized and defined, will achieve the goals of the RDoC initiative, namely to focus on disturbances of specific brain functions to better understand the underlying causes of mental disorders. We argue that the attempt to define language as a distinct unitary RDoC construct results in a construct that is both too narrow but also too broad. One alternative strategy involves deconstructing language into sub‐constructs for study. Although a comprehensive evidence‐based taxonomy of language that successfully integrates behavioral and neurobiological features does not exist at this time, linguistic, speech, communication, and cognitive sciences have made strides in demarcating distinct subcomponents of language. A handful of salient and highly replicable findings emerge from this literature that can be used to inform how language could be organized using an RDoC structure.

Consider one of the most well‐regarded findings from neurolinguistics involving the neuroanatomical distinction between language perception (e.g., involving Wernicke's area) and language production (e.g., Broca's area) [Lezak, 2004; Strauss et al., 2006]. Functionally speaking, damage to these regions results in distinct aphasias; though manifest across modes of communication (e.g., verbal, visual sign language). Neurodevelopmentally speaking, receptive and expressive language delays are distinct, with highly similar prevalence rates and only modest overlap across the population [Law et al., 2000]. Thus, it is clear that that perceptual and production abilities are functionally and neurobiological distinct, and should be considered as separate entities within the RDoC construct of language.

Even these linguistic subdomains are overly broad. Consistent throughout modern linguistic theories is the notion that language is composed of separable components (e.g., Levelt, 2013). Interestingly, variability in these components explains how languages differ across cultures and dialects. These components include the following: (i) phonetics and phonology—referring to the way in which language is expressed and perceived through sounds and signs, and the way in which these sounds and signs are organized into systems (ii) prosodics—referring to the way in which stress, intonation, rhythm, speaking rate, or voice quality complement, and modulate meaning of language, (iii) syntax and morphology—referring to the internal structure of how words and phrases are organized, (iv) semantics—referring to the conceptual meaning of language, and (v) pragmatics—referring to the knowledge of when and how to use language, and how to do things with words [Warren, 2012]. These components are each important to both the perception and the production of language, while abnormalities in these components differentially correspond to distinct symptoms of mental illness. For example, disruptions in prosodic but not necessarily semantic or syntactic aspects of speech are characteristic of psychomotor retardation in depression and blunted affect in schizophrenia [Andreasen, 1984; Cohen et al., 2012; APA, 2013]. Similarly, aberrant semantic communication is a hallmark of tangential speech in schizophrenia; speech that is often phonetically, prosodically, and syntactically unremarkable [Andreasen, 1981; APA, 2013; for a review see Elvevåg and Goldberg, 1997].

**Figure 1:**
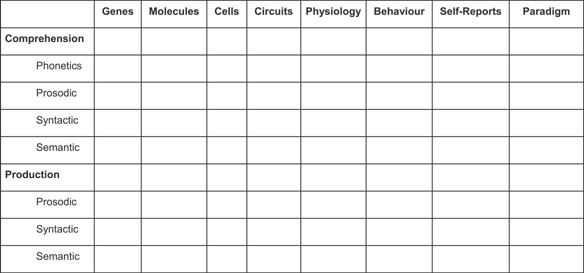
A preliminary organization of language for RDoC.

An initial attempt to expand the language construct for RDoC is highlighted in Figure 1. By no means is this figure comprehensive, though it does employ well‐accepted constructs that can serve as a starting place until more scientifically useful replacements can be realized. Functionally speaking, an organization involving these constructs while accounting for pragmatics and basic cognitive abilities can provide critical information about mental illness and their mechanisms and can be illuminated by the use of relatively novel highly sensitive technologies.

## THE FUTURE OF LANGUAGE MEASUREMENT IN AN RDoC FRAMEWORK

Three major innovations promise to reshape how language, particularly production subdomains, will be assessed. These include the development and application of novel technologies, novel analytic procedures, and big data and large‐scale biobanking efforts.

### Innovation 1: Novel Technologies

A first major innovation involves the development and application of relatively inexpensive technologies for understanding key language functions, for example, the use of portable acoustic, lexical, and semantic analysis [Cohen and Elvevåg, 2014]. While many of these technologies have existed for decades, the scientific knowledge base supporting their use has improved in recent years, and their application across disciplines has helped resolve obstacles limiting their implementation. For example, crowdsourcing has been used to provide larger data sets for statistical speech and language processing [e.g., Novotney and Callison‐Burch, 2010]. Hardware advances, for example, using ultrasound assessment of physical vocal processes, also have been developed. Ultrasound is safe [Epstein, 2005], non‐invasive, and has been used in linguistic fieldwork [Gick et al., 2005] and speech therapy [Bernhardt et al., 2005]. It can, thus, easily be applied to psychiatry research. Technological advances, such as smaller sensors and improved signal processing, allow the relatively unobtrusive collection of continuous data, while a patient navigates their daily routine, thus extending assessment well beyond the confines of the clinical setting and providing naturalistic data. Key innovations are highlighted in the following section.

#### Measures of speaking rate

One key example in the use of these novel technologies is in the analysis of speaking rate. As discussed above, language use is heavily affected by context. This means that in order to determine whether a particular language‐related indicator is indicative of mental illness, and might be linked to a relevant behavioral phenotype, we first need to establish whether the variation might not be due to other factors. A case in point is speaking rate, that is, the speed at which a person produces words, syllables, and sounds. Slowed speaking rate is one of the key indicators of depressed mood [Cannizzaro et al., 2004; Mundt et al., 2012; Cummins et al., 2015], and is thought to be related to general psychomotor retardation, which also affects measures such as reaction time [Sobin and Sackeim, 1997; Buyukdura et al., 2011; Bennabi et al., 2013]. Speaking requires a person to coordinate the movement of jaw, lips, vocal folds, tongue, velum, and (depending on the language] uvula with millisecond precision [Laver, 1994]. It is a highly skilled process that can be disrupted both at the level of planning (apraxia of speech) and execution [dysarthria; Duffy, 1995]. In clinical neurology, slowed speech often occurs together with general fine and gross motor retardation [Duffy, 1995].

While it may appear tempting to use an appropriate absolute measure of speaking rate (fast/medium/slow) as part of a behavioral phenotype relating to psychomotor retardation, such an approach would introduce gross distortions. Speaking rate has been shown to vary by language [Yuen et al., 2006], dialect [Jacewicz et al., 2009], and age [Benjamin, 1997; Jacewicz et al., 2009; Mefferd and Corder, 2014]. As the overview by Cummins et al. [2015] shows, most of the studies that did find significant effects of depressed mood on speaking rate were pre/post‐studies that tracked the same population during treatment. Therefore, the appropriate behavioral marker is not speaking rate as such, but change in speaking rate over time. Similar caveats hold for most of the phonetic and phonological behavioral markers of language that might reflect mood, and we argue that these measures should be ascertained longitudinally in large biobanking efforts.

#### Acoustic and prosodic measures

Prosodic production, characterized by patterns of speech rhythm, vocal fold vibration frequency, and volume in vocal expression, is an important nonverbal facet of communication and is affected in a number of psychiatric disorders such as depression, schizophrenia, and autism‐spectrum disorders [Andreasen, 1984; Cohen et al., 2012; APA, 2013]. Using clinical rating scales in schizophrenia samples, for example, these deficits are estimated to be on the order of three to five standard deviations below nonpsychiatric populations [Cohen et al., 2014a]. While these deficits are assumed to be stable over time, it is not clear that they are static over context.

Generally, a number of contextual variables influence prosodic production, for example, emotional [Tolkmitt and Scherer, 1986; Sobin and Alpert, 1999], arousal [Johnstone et al., 2007; Cohen et al., 2010], and social [Nadig et al., 2010] factors, to name a few. Emerging results have been taken to suggest that prosodic production is also linked to cognitive state variables. Of note, a number of studies, mostly correlational in nature, have documented links between acoustic properties of natural speech and state measures of cognitive stress, for example, in how vocal expression in air pilots changes as a function of demanding flight conditions [e.g., Huttunen et al., 2011].

More generally speaking, cognitive load [Plass et al., 2010] and information processing [Baddeley, 1992; Tombu et al., 2011] theories suggest that resources for engaging in motivated activities/behaviors are finite, and thus reflect a “bottleneck” for central nervous system operations more generally [Tombu et al., 2011]. When this capacity is exceeded, either because of task complexity or demands from competing tasks, performance is impaired. Indeed, increased processing load is associated with reduced performance on a range of learning, motor, and other activities [e.g., Plass et al., 2010] within healthy adults. This result has been important for understanding illness state for a broad range of neurological and psychiatric conditions, such as Alzheimer's disease [Huntley and Howard, 2010], various dementias [Calderon et al., 2001], and schizophrenia [Granholm et al., 2007].

If indeed prosodic deficits manifest, at least in part, as a function of limited cognitive resources, then several important and clinically‐pertinent implications warrant mention. From an assessment perspective, acoustic analysis of natural speech may provide insight into an individual's cognitive functioning or mental state more generally. Thus, longitudinal tracking of individuals who are either experiencing or at risk for experiencing cognitive difficulties, for example, in older adults experiencing mild cognitive impairments or individuals at‐risk for psychosis, may provide valuable information about their clinical state and treatment needs, especially within the context of large biobanking efforts. Notably, prosodic production was found to be an important biomarker in a recent longitudinal study of mild cognitive impairment and dementia [Satt et al., 2014].

Importantly, acoustic vocal analysis is easy to conduct, repeatable and objective in a way traditional clinical assessments are not. Assessment of natural speech offers many practical advantages over standard neuropsychological tests, for example, employing data capture over mobile technologies, and has the potential to detect subtle changes in information processing capacity in a way not practical with standard neuropsychological measures [see Mundt et al., 2007; Cohen and Elvevåg, 2014 for elaboration]. Standard clinical language measures (e.g., verbal fluency) suffer from profound practice effects even after a few administrations [Lezak et al., 2012]. From a treatment perspective, prosodic deficits may ameliorate by improving cognitive resources more generally; by employing cognitive compensation strategies (e.g., limiting activities requiring multi‐tasking) or by bolstering capacity or efficiency more generally (e.g., cognitive remediation). While admittedly in its infancy in terms of psychometric evaluation, vocal analysis offers promise as a window into more basic cognitive operations.

### Innovation 2: Novel Analytics

#### Natural language processing and machine learning

A second innovation involves the advancement of statistical natural language processing and machine learning techniques applied to genetics and clinical cognitive neuroscience [e.g., Hofmann, 2001; see also Nicodemus and Malley, 2009; Cohen et al., 2014b]. On the genetics side, these approaches have successfully detected validated epistasis in schizophrenia [Nicodemus et al., 2010a,b, 2014b].

In the last two decades, a variety of lexico‐semantic modeling approaches have gained popularity within cognitive science and subsequently within cognitive neuroscience and clinical science. One such cognitive modeling approach that has gained increased attention is latent semantic analysis (LSA), which uses natural language processing techniques to extract word meaning from text [Furnas et al., 1988; Deerwester et al., 1990], and it has been heralded as a theory of meaning [Landauer, 2007] and a computational model of vocabulary acquisition [Biemiller et al., 2014]. The major idea behind these models is that people are sensitive to weak statistical regularities in the linguistic environment, such as the co‐occurrence of words in a sentence. Using text corpora, LSA can learn the meaning of a word by estimating the relatedness of any arbitrary set of words as a function of the contexts in which they co‐occur. One of the key advantages of this approach is that, using singular value decomposition or probabilistic inference, representations for all kinds of words including abstract or low frequency words can be derived, even if those words never co‐occur in the same text or sentence. Large, corpus‐based statistical models of language have enabled the operationalization of semantic structure of discourse because they in essence quantify semantic similarity by analyzing large sets of documents. Related techniques include Topic Models [Blei et al., 2003], Independent Component Analysis [Hyvärinen et al., 2004], and Neural Networks, specifically Deep Learning [Hinton et al., 2006].

Evidence for the success of many of these approaches derives from solving difficult problems in computer science, such as speech recognition and image annotation (in the case of deep learning), rather than a focus on simulating and understanding cognition per se. Put differently, although at first glance, it might be tempting to assume that these methods simulate human data so well because at their core are basic low‐level functions that may be somewhat analogous to neurocognitive processes in the brain during learning and memory formation; however, this is most probably not the case. Rather it may be that such techniques are sensitive because they accurately model the structure of meaning as imposed by the limits of brain function averaged across large quantities of text. Thus, when presented with examples of discourse that are “different,” it is because the language originated from a person where divergent development or injury has changed the boundaries imposed by brain function and we can detect those differences. Thus, although it is possible that LSA may in fact be model of cognition, an alternative possibility is that it has just learned some of the structure of semantics that the brain imposes on human composed text, and other learning algorithms might yield similar results. Therefore, it may not be surprising that such models might be more sensitive than either humans or simple measures over text (Mark Rosenstein, personal communication, June 2015).

Natural language processing and machine learning techniques provide much needed and necessary tools to re‐define what we understand by language and how it can be usefully studied within neuropsychiatry. In addition, these fields provide methodologies for combining different types of high‐dimensional data (e.g., speech and neuroimaging, genomics and speech, genomics and clinical data), using ensemble machine learning methodologies such as mixture‐of‐experts approaches [Jacobs et al., 1991; Jordan and Jacobs, 1994; Lê Cao et al., 2010]. Successful examples of these innovations applied to SMI research include predicting from discourse samples who among those at risk will eventually transition to psychosis [Bedi et al., 2015]. This result expands on recent similar computational language approaches that use discourse alone to successfully discriminate patients with schizophrenia from controls [Elvevåg et al., 2007], discriminating schizophrenia probands, first‐degree relatives, and unrelated healthy controls [Elvevåg et al., 2010], and differentiating those at high risk of psychosis from unrelated putatively healthy participants [Rosenstein et al., 2015].

It seems realistic to anticipate that these machine learning and natural language processing approaches will provide the foundation for the much needed new language phenotypes that is at the core of the RDoC mission. However, in order to achieve this enormous potential that these approaches afford, we must strive to collect purpose‐designed data sets, containing large populations on which the power of natural language processing techniques can be leveraged so as to ensure effective assimilation into clinical research to provide valid and reliable measures [Foltz et al., 2016]. This broader and more modern definition of language (i.e., that goes beyond previous work that focuses on simple aspects such as word count) can shed light on their genetic and neurobiological mechanisms and concomitants and offer insight into how language can go awry in psychiatric and neurological disorders.

#### Computational network sciences

Computational network sciences tools have also recently been applied to study the mental lexicon. Uses include attempts to shed light on the putative rigidity of thought in those with Asperger's versus healthy controls [Kenett et al., 2015], to examine creative thinking [Kenett et al., 2014], to chart thought disorder in patients with psychosis [Mota et al., 2012], and to detail the effect of drugs of abuse (MDMA [“ecstasy”] and methamphetamine) on spoken language [Bedi et al., 2014]. Indeed, network‐based models of cognition offer a multi‐level research approach where the global (macro), intermediate (meso), and detailed (micro) structure mutually constrain processes and representations [Baronchelli et al., 2013]. Such network approaches to the mental lexicon provide an extension of the classical model proposed by Collins and Loftus [1975] as well as an alternative to the notion of a hierarchical taxonomic knowledge repository. Also, since these models can include the majority of words used in language the structure is primarily thematic in nature [De Deyne et al., 2015a,2015b]. The sheer scale of these networks provides metrics sensitive to the dynamic processes in language production, as well as the structural ones. Such approaches are well‐suited to looking at individual differences either at a case level or group level, and promise to be of great value in charting the mental lexicon in patients with severe mental illness as compared with a normative network [De Deyne et al., 2015c]. Such rich network approaches thus enable the examination—within a single framework—of factors such as the degree of organization and efficiency of information retrieval, type of information that is activated in language use as well as the accessibility of words. Importantly, given the highly interdependent nature of each level, possible interpretations are naturally constrained. These network approaches illustrate the emerging possibilities of combining a modern understanding of language with unprecedented computational facilities to create a new research framework for objective investigations that can establish the locus of aberrations in a dynamic network. The combination of these linguistic computational approaches with state‐of‐the art machine learning applied to genomics, neuroimaging, or other high‐dimensional omics data will lead to advances in better understanding SMI, and, ultimately, the goals of personalized medicine.

### Innovation 3: “Big Data” Applications and Large‐Scale Biobanking Efforts

A final innovation involves the integration and synthesis of large scale data collection efforts such as that undertaken by Generation Scotland [Smith et al., 2006] or the UK Biobank [Collins, 2011]. A major result from the machine learning community is that big data is at least as important as algorithms in achieving high performance in machine learning tasks. Recently, Google released an open‐source machine learning infrastructure TensorFlow, and as noted in Technology Review, “Google was able to give away the code for TensorFlow because the data it owns is a far more valuable asset for building a powerful AI engine.” [Knight, 2015]. For many of the approaches described above, big data are critical for learning models and validating hypotheses.

The collection and curation of natural language samples has been a staple of communication sciences for decades. These databases of samples (also called corpora) are the linguistic equivalent of biobanks [Biber et al., 1998]. While some corpora are domain specific (e.g., the Canadian Hansard parliament transcripts, http://www.isi.edu/natural-language/download/hansard/), others strive to collect samples from a range of communication contexts (e.g., the British National Corpus of UK English, http://www.natcorp.ox.ac.uk). The material in these corpora ranges from written to spoken language, from read to spontaneous speech, from conversations to radio news, and from video‐recorded meetings to novels. However, application of these large databases to understanding SMI has been limited. In the last 20 years, as part of research on the automatic detection and generation of emotional speech, corpora have been created that contain information about the mood of the speaker or writer. For example, as part of ongoing, long‐term work on the detection of emotion in speech, the speech technology community has been creating data sets such as the corpus that was used for the AVEC 2013 Depressed Speech Challenge [Valstar et al., 2013], which features both video and audio data, and contains speech that has been annotated with mood.

Another source of big data for language research is the mining of microblogging (e.g., Twitter and Facebook posts). Research on microblogging platforms may be limited to evaluating only certain aspects of language; however, it can provide important information about lexical expression. Research of this kind is still in its infancy, but is providing potential sources of longitudinal data for text and emoticon mining, including data pre‐dating an acute episode of SMI and during the recovery phase. Using machine learning of case status from Twitter posts of self‐reported cases of schizophrenia versus healthy controls showed high precision (92%) and moderate recall (71%) in predicting case status on independent, “held out” test data. The features that were most important in predicting case versus control status were the use of the word “schizophrenia”, increased happy emoticon usage, and timing of posts, especially more frequent early morning posts [McManus et al., 2015]. An earlier study also reported 70% accuracy and 74% precision using Twitter posts preceding the onset of a Major Depressive Episode in individuals self‐reporting being clinically diagnosed with MDD [De Choudhury et al., 2013]. Critical predictors of a Major Depressive Episode that could be obtained via Twitter posts included increases in negative affect, social/medical concerns, the closeness of social networks, and religious involvement; this was accompanied by decreases in social engagement. These types of microblogging data resources may be combined with large‐scale biobanking efforts as a source of longitudinal data to provide data pre‐dating ascertainment for these cohort studies.

One major limitation in the use of biobanking for understanding the genetics of language disorder is that the heritability of language abilities more generally is poorly understood. Proof of heritability—that a trait is genetically influenced—is required before the next logical step of determining its genomic architecture. The collection of data from these novel technologies and application of novel analytics can provide both the foundational heritability estimates and the large‐scale normative data required for understanding differences among those with SMIs. For the phonetic and prosodic levels, objective, robust measures are needed that focus on longitudinal trends, as many of the key behavioral signs are subject to age, dialect, and sociolinguistic variation. Since speakers adapt their language production to their interlocutors, and the setting in which they speak, such longitudinal measures need to be obtained under comparatively controlled circumstances, as well as establishment of reliably and validity of the metrics. In order to be able to leverage big data approaches, we need innovative ways of collecting data that provide regular samples of speech data from the same individual over the course of months to years, ideally covering at least one cycle of illness and recovery.

### CLOSING REMARKS

In many ways, the oversimplification of language in the current version of the RDoC matrix is a function of the inadequacy of historical‐based definitions of language, and consequently the measures that were traditionally used to measure it, such as verbal fluency tasks which tap only very basic and circumscribed linguistic processes. However, recent technological advances in mobile telephony, multimedia assessment, ubiquitous computing, ultrasound, statistical modelling, and neuroimaging afford previously unimaginable opportunities to collect remarkably rich, high‐dimensional naturalistic data that provide hitherto untapped information about people's ability to comprehend and use appropriate language in context, the fine‐grained temporal detail regarding articulatory gestures, and assays of the effectiveness of their linguistic communication. As the data collection methods continue to evolve in terms of their resolution, so too do the data analytic methods. Computational linguistic, cognitive, affective, and speech scientists have already devised impressive data mining and analytic methods, such as latent semantic analysis and lexical analysis, to make these high‐dimensional datasets tractable via dimension reduction, information theory, and statistical machine learning techniques. These analytic methods continue to evolve in sophistication so as to better leverage the evolving levels of resolution in the data. This trend is fundamentally important as analysis of language holds enormous (mostly untapped) value for understanding cognitive, affective, physiological, and pathological states more generally and can serve as a proxy of brain health. In this article, we have argued for a broader definition of language and one that is motivated by modern cognitive neuroscience and which usefully reconceptualizes language processes such that they can be of value in translational research and thereby shed light on critical issues in the NIMH RDoC initiative. In turn, the combination of computational linguistic approaches, natural language processing, and machine learning in genomics and neuroimaging—and combining data across these high‐dimensional data types—will lead to significant advances in understanding SMIs and pave the road toward personalized medicine.
